# Editorial: Rhythm across the arts and sciences: a synergy of research

**DOI:** 10.3389/fpsyg.2023.1188121

**Published:** 2023-04-20

**Authors:** Adina Mornell, Bettina E. Bläsing, Frank Heuser, Horst Hildebrandt

**Affiliations:** ^1^Instrumental and Vocal Music Education, University of Music and Theater Munich, Munich, Germany; ^2^Department of Rehabilitation Sciences, Technical University Dortmund, Dortmund, Germany; ^3^The Department of Performance, Music Education, and Composition in the UCLA Herb Alpert School of Music, University of California, Los Angeles, Los Angeles, CA, United States; ^4^Music Physiology and Musicians' Medicine, Zurich University of the Arts, Zurich, Switzerland

**Keywords:** music, psychology, rhythm, neuroscience, perception

Readily apparent in music and dance, the universality of rhythm and rhythmic processes also permeates art, architecture, sports, neuroscience, psychology, and medicine. This collection presents multiple perspectives from these diverse domains that challenge assumptions and offer new insights into the pervasiveness and trans-disciplinary nature of rhythm and its centrality to the human condition. Rhythm is an essential part of art and life, found in music, movement, circadian cycles, and learning. The predictable patterns of regularity that exist throughout the physical universe and in human activities enable us to understand the world, generate meaning, and engage in both quotidian as well as inspired creative activities.

Although one might expect the majority of articles in a collection about rhythm to revolve around music, four papers look at different art forms: dance, poetry and visual arts. First, Wilson and Henley investigate the thoughts of three generations of dance teachers about the experience of rhythm, from Margaret H'Doubler's teaching about rhythm as “measured energy” to Wilson and Henley's research work on movement qualities, and further to Henley's educational approaches for dance students to explore and experience rhythm pre-reflectively.

Stupacher et al. aim to elucidate the neural mechanisms underlying groove, which was defined as the pleasurable urge to move to a rhythm. They look at the relationship between predictability and surprise in the experience of groove. Their study covers dance, music making, and music listening, in search of a better understanding of the interactions between temporal processing, movement, social behavior, and pleasure. The paper also discusses implications for future research that could encompass treatments for patients with motor impairment (e.g., Parkinson's).

In contrast to dance, poetry is at the center of the Beck and Konieczny study which investigates top-down and bottom-up processes while reciting poems out loud. The rhythmicity of the reading was modified when the syllable “tack” replaced randomly selected syllables. Participants were recorded reading both original and manipulated versions of the poem. Results of the analyses of syllable duration and intensity suggest that processes from both directions interact, and that the top-down processes supporting the metric structure seem stronger in participants with musical experience.

Hadavi et al., on the other hand, examine the positive effects of viewing visual artworks on visitors' mood and wellbeing. In a study conducted during the pandemic, participants visited a virtual exhibition of works, which had been created in response to specific musical compositions. The researchers did not find differences in mood when comparing those participants who viewed the artworks while simultaneously listening to the associated musical compositions and those who saw the art without sound. They demonstrate that a virtual art exhibit was able to increase positive affect and decrease negative affect in a widespread audience.

Other papers look at internal, biological and physiological phenomena. In their article, Kwak et al. describe a trio model of human biological rhythms—central rhythms, internal/external rhythms, and reflex/consequential rhythms—and their cycles. The authors view these three types of biological rhythm as members of a musical ensemble, which, in flexible mutual exchange and interconnection, lay the foundation for homeostasis as well as regeneration.

Through a single case study, Sebastiani et al. describe the influence of technical challenges, temporal, and emotional factors on heart rate variability in piano playing. When playing a classical piece, increased activation of the sympathetic nervous system was found, in contrast to when they played a jazz piece. This was presumably caused by challenges with regard to precision and correct playing technique. These challenges, in turn, appear to influence temporal features and emotional involvement.

Evidence exists that rhythm-based activities can be helpful for those who have experienced trauma by regulating arousal levels and supporting positive experiences. Building upon this, McFerran et al. use an action-based approach to investigate young people's responses to music therapy treatments after traumatic experiences. According to the data collected, participants preferred semi-structured activities that allowed for creativity, self-direction and individuality, and enjoyed moments of co-regulation and matching their own rhythms rather than entraining to an external rhythm.

Rhythm objects are defined by Godøy as “strongly coherent chunks of combined sound and body motion in music” in a literature review that looked at music psychology, music theory, philosophy and research in human movement science. In this article, the author considers rhythm beyond traditional and conventional definitions. He describes chunks of musical sound and the bodily motions associated with their production, that could be perceived holistically as quantal elements in musical experience.

Margulies et al. investigate the close temporal relationship between technical demands and compensatory movements of the left upper extremity while playing the violin. Minimizing compensatory movements could help violinists avoid playing-related health problems. Strategies discussed include individually optimizing the position of the instrument, frequently changing practice strategies when working on challenging passages, and taking breaks more often during practice sessions.

Honda and Fujii focus on the differences between amateur and professional darbuka players while learning rhythmically challenging finger tapping movements. Professionals showed better learning results both in terms of speed and precision of the movements, which the authors attribute to their greater wealth of experience with the instrument. Consistent with the efforts in music, dance, and sport, documented since 2008 in the framework of the *Art in Motion* symposia, these results suggest a need for optimization of instrument-related learning and practice strategies.

Taken together, this collection presents diverse and interdisciplinary research, all based on the common theme of “rhythm.” These ten articles span a broad variety of topics, by looking at phenomena through studies of music-making, perceptual processing, as well as other aspects of human biology and behavior. The editors hope that these articles will serve to extend and challenge our conceptions of rhythm, and suggest questions for further research.

## Author contributions

All authors listed have made a substantial, direct, and intellectual contribution to the work and approved it for publication.

